# Appearance of Unstable Monopoly State Caused by Selective and Concentrative Mergers in Business Networks

**DOI:** 10.1038/s41598-017-05362-5

**Published:** 2017-07-11

**Authors:** Hayato Goto, Eduardo Viegas, Henrik Jeldtoft Jensen, Hideki Takayasu, Misako Takayasu

**Affiliations:** 1 Department of Computational Intelligence and Systems Science, Interdisciplinary Graduate School of Science and Engineering, Tokyo Institute of Technology, 4259, Nagatsuta-cho, Yokohama, 226-8502 Japan; 2 Centre for Complexity Science and Department of Mathematics, Imperial College London, SW7 2AZ London, United Kingdom; 3 Institute of Innovative Research, Tokyo Institute of Technology, 4259, Nagatsuta-cho, Yokohama, 226-8502 Japan; 4 Sony Computer Science Laboratories, 3-14-13, Higashi-Gotanda, Shinagawa-ku, Tokyo, 141-0022 Japan

## Abstract

Recently, growth mechanism of firms in complex business networks became new targets of scientific study owing to increasing availability of high quality business firms’ data. Here, we paid attention to comprehensive data of M&A events for 40 years and derived empirical laws by applying methods and concepts of aggregation dynamics of aerosol physics. It is found that the probability of merger between bigger firms is bigger than that between smaller ones, and such tendency is enhancing year by year. We introduced a numerical model simulating the whole ecosystem of firms and showed that the system is already in an unstable monopoly state in which growth of middle sized firms are suppressed.

## Introduction

The analysis and modeling of our society is providing profound new insights such as cascading failures^1^ and resilience^2, 3^ using the lens of the science of complex systems^4–7^. In earlier research on a national network of transactions between businesses, which can be regarded as a typical real world example of a complex network having both the basic properties of small world^8^ and scale-free^9^ accompanied with various scaling relations^10–18^, it was found that the balance between new entry, bankruptcy and merger plays the key role in the time evolution of this system^19, 20^. Here, we focus on the recent comprehensive data of Mergers and Acquisitions (“M&A”) in Japan, more than 41 thousands events, and find that the network evolution is essentially unstable due to two basic inherent business dynamics; the preferential selection, whereby acquiring companies tend to be bigger than their targets, and concentrative M&A, in which the probability of merger among bigger companies is higher. As a result, the time evolution equation with the parameters estimated from recent data indicates that there is no stable steady solution in the size distribution of firms. Our numerical simulation shows that the selective M&A mechanism leads to significant concentration resulting in the appearance of business monopolies and oligopolies, akin to those types described in other research such as super connected companies^21^. Importantly, such companies suppress growth of middle size firms, and therefore suppress the emergence of new challengers that are fundamental to the functioning of an ecosystem. Sooner or later when the giant fails, a turbulent period follows until another giant appears repeating with a typical time scale of a few tens of years.

## Results

### Increasing of M&A activities

**Figure 1:**
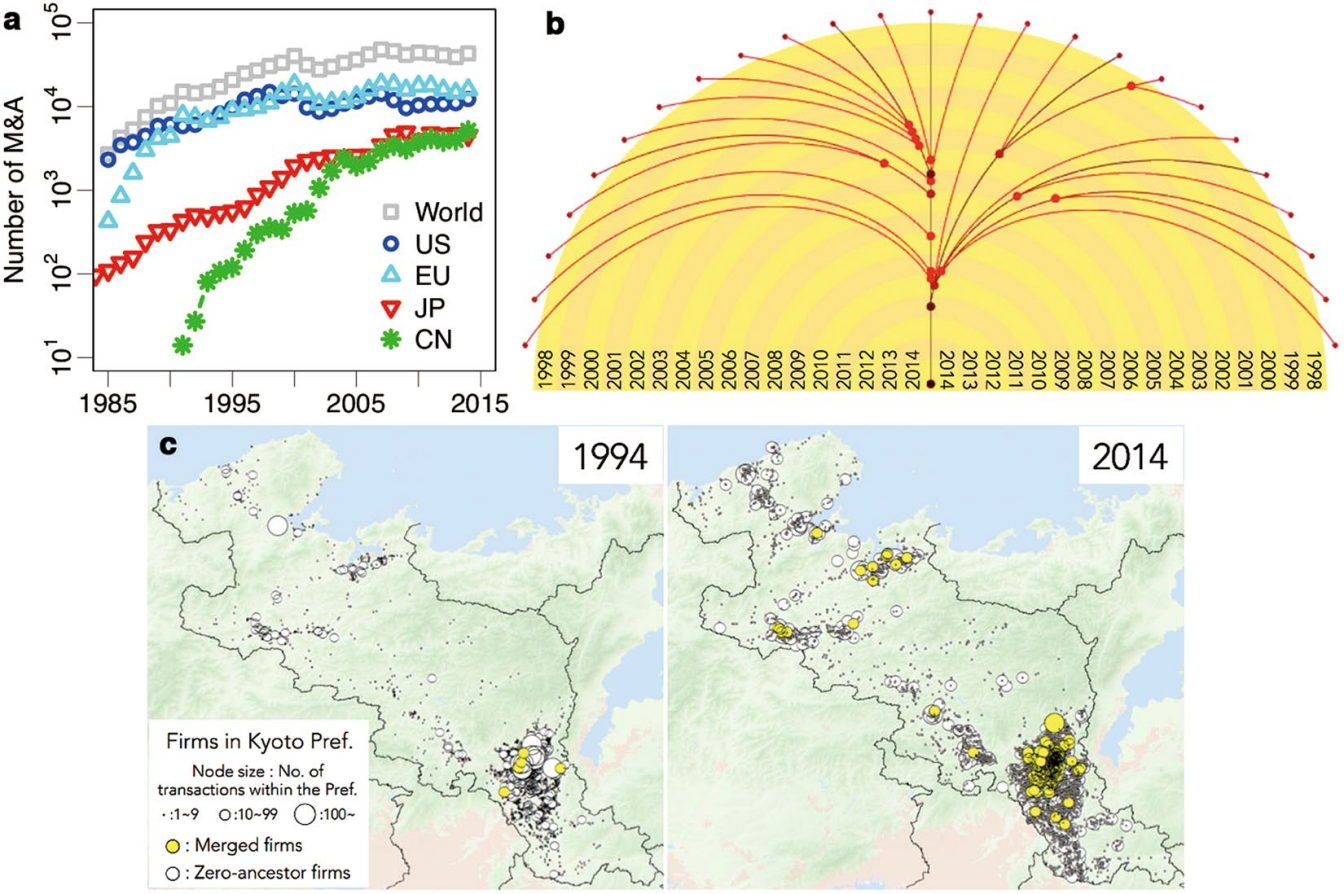
Increasing of M&A activities. (**a**) Change of the annual number of mergers and acquisitions on a semilog scale. Each plot shows the evolution in numbers of M&A for the whole world (gray), US (blue), EU (light-blue), JP(red) since 1985 (red), and CN since 1988 (green). (**b**) An illustrative example of an actual firm merger history diagram (Generated by MATLAB 8.5 software^30^). Each node within the colored region relates to a single company merger within the year indicated by the corresponding semi circled color strip, whereas those out of the boundary represents each single company prior to 1998. Each arched line represents the period of existence of a given company since 1998,whereas the central vertical line corresponds to the surviving company. (**c**) Spatial distribution of firms that are sized by number of transaction partners within Kyoto Prefecture in 1994 (left) and in 2014 (right) (Generated by Arc GIS 10.2 software^31^). Yellow and white nodes are firms that have merged or not, respectively. In addition, the larger the number of transaction partners a firm has, the bigger the radius of node.

According to comprehensive business firms data (see Supplementary Information; Data source), the annual number of M&As in Japan in the last 30 years has increased more than 10 times as in other countries such as the EU and China in Fig. 1a (all data of the whole world, the US, the EU and China are sourced from the Institute for Mergers, Acquisitions and Alliances). This tendency is clearly illustrated by an actual merger history diagram of a particular firm, which is the center position node (Fig. 1b). M&A activity has a potent influence on business transactions and hence is one of the most important corporate activities. We next focus on inter-firm transactions network aspect of M&A. Figure 1c shows the spatial distribution of firms sized by number of transaction partners, or link number, *k* within Kyoto Prefecture in 1994 and 2014. In this case, it appears that majority of firms with high link number in 2014 have been involved in mergers (yellow circles), although it is equally noticeable that the majority of firms in 1994 had no merged ancestors (white circles).

### Scaling behaviour and power laws

It has been shown mathematically that M&A activity is one of the main causes of the power law link number distribution of firms with exponent 1.4 measured by *k* ^19^. Firm sizes can equally well be measured by number of employees *E*, by annual sales *S*, and number of ancestors *A* correlates with number of employees and annual sales. There are scaling laws between pairs of *k*, *E*, *S*, and *A* such that $S\propto {k}^{1.3}$, $S\propto {E}^{1.3}$, $E\propto {k}^{1.0}$, $k\propto {A}^{1.4}$ (Supplementary Fig. 2), and we apply *k* as the measure of firm size in the following discussion (it can be substituted *E*, *S* or *A* given that there are similar scaling laws among those quantities as previously described). Moreover, all these quantities follow power law cumulative distributions with exponents 1.4, 1.0 and 2.4, respectively (Supplementary Fig. 1).

### Statistical characteristics of M&A activities

**Figure 2:**
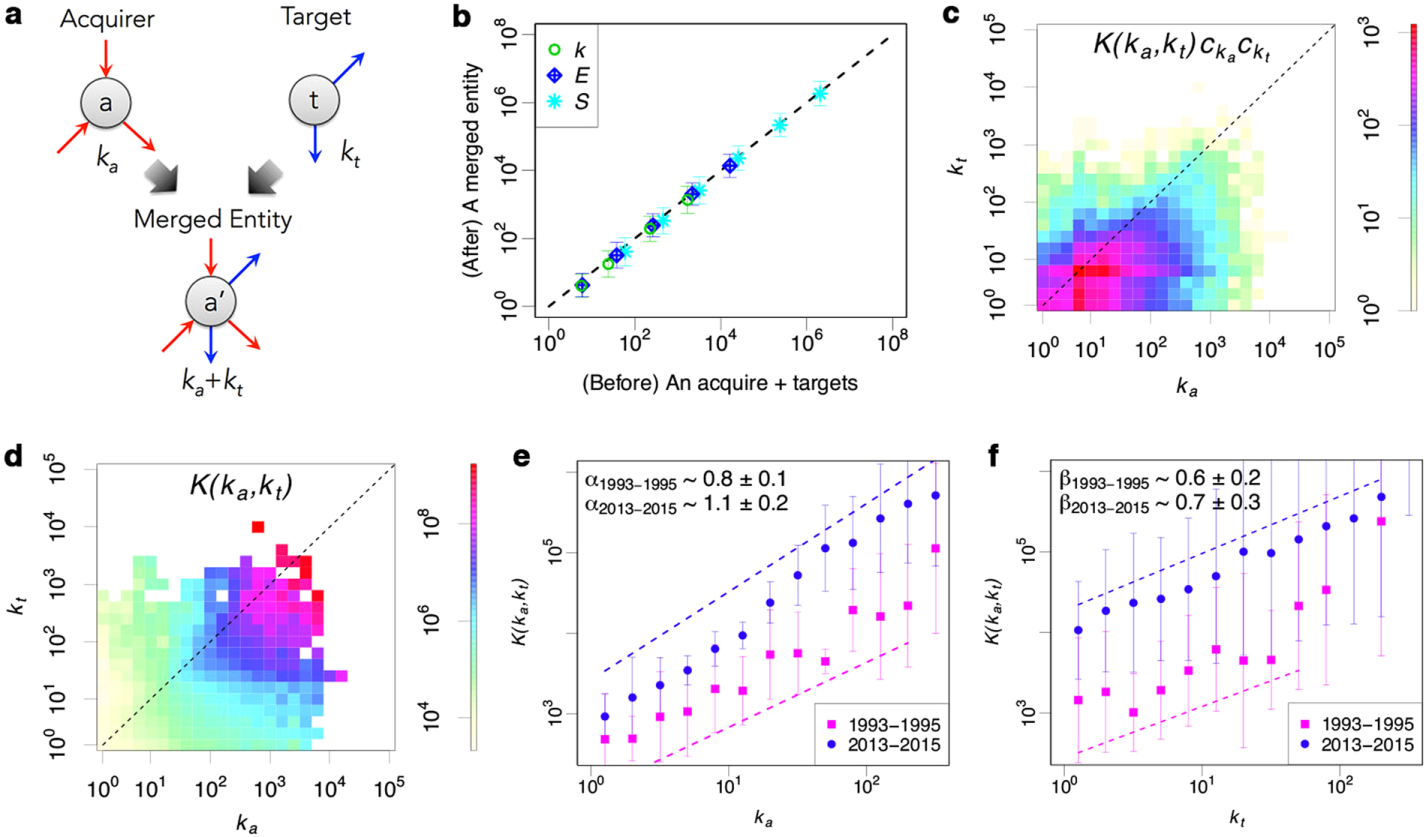
Statistical characteristics of M&A activities. (**a**) A schematic figure showing a merger event. (**b**) The relation between a size of merged entity and summation of sizes of an acquirer firm and target firms in 2014. Green circles, blue rhombuses and light blue asterisks show the mean number of transactions *k*, number of employees *E* and sales (million yen) *S*, respectively. (**c**,**d**) Heatmap showing the frequency and probability of M&A between an acquirer firm of size *k*_*a*_ and its target of size *k*_*t*_ based on the data from 1994 to 2014. (**e**,**f**) *K*(*k*_*a*_, *k*_*t*_)’s scaling laws with respect to *k*_*a*_ and *k*_*t*_, respectively. Pink squares and blue circles show the relation between the mean of *K*(*k*_*a*_, *k*_*t*_) and *k*_*a*_ or *k*_*t*_ with fixed *k*_*t*_ or *k*_*a*_ from 1993 to 1995 and from 2013 to 2015 in log-log scale, respectively. Each equally color coded dashed-line shows a slope of *α* and *β*.

By making use of the data, we are able to determine the statistical characteristics of firms M&A activities. Firstly, we compare a merged entity with the sum of an acquirer firm *a* and target firms *t* (Fig. 2a). As a result, we find that M&A almost keeps the conservation law for each volume such as *k*, *E* and *S* (Fig. 2b and Supplementary Fig. 3). Based on the simple law, we can draw inspiration from Smoluchowski coagulation equation^22^ to observe a frequency of merger $K({k}_{a},{k}_{t}){c}_{{k}_{a}}(\tau ){c}_{{k}_{t}}(\tau )$ and merger kernel *K*(*k*_*a*_, *k*_*t*_). Note that *k*_*a*_ is a link number *k* of an acquirer firm *a*, and *k*_*t*_ is that of a target firm *t*, and *c*_*k*_(*τ*) is a probability density of firms with *k* in time *τ*. We then observed $K({k}_{a},{k}_{t}){c}_{{k}_{a}}(\tau ){c}_{{k}_{t}}(\tau )$ (Fig. 2c) and *K*(*k*_*a*_, *k*_*t*_) (Fig. 2d). With regards to the process of mergers and acquisitions among firms, we identify two key characteristics;

1. Acquirers tend to be bigger than their targets and to have a preference (Fig. 2c).
2. The probability of merger between bigger firms is higher than that between smaller ones (Fig. 2d).

We simultaneously find the *K*(*k*_*a*_, *k*_*t*_)’s scaling laws with *k*_*a*_ and *k*_*t*_ (Fig. 2e,f) as follows;

1 $$K({k}_{a},{k}_{t})\propto {k}_{a}^{\alpha }{k}_{t}^{\beta }$$

Here, $(\alpha \mathrm{,\ }\beta )\simeq (0.8\pm 0.1,\mathrm{\ 0.6}\pm 0.2)$ from 1993 to 1995, and $(\alpha \mathrm{,\ }\beta )\simeq (1.1\pm 0.2,\mathrm{\ 0.7}\pm 0.3)$ from 2013 to 2015. We make a distinction between two separate elements of the M&A process as ${k}_{a}^{\alpha }$ corresponds to the willingness of a company to merge, whereas ${k}_{t}^{\beta }$ is the choice, or the selection, of a given partner. We observed these exponents by the least squares method with coefficient of determination *R* ^2^ ≥ 0.9 and the error-bars show standard deviation. That is, these exponents characterize the tendencies toward M&A activity, which has statistically significantly increased recently. We note that we have accumulated 3 years of data (1993–1995; 2013–2015) in order to accurately estimate the statistical properties of the merger kernels of 1994 and 2014 by increasing the sample sizes. Besides, as for the established firm case in 1994 and 2014, we can observe certain level of preferential attachment^9, 19^. Supplementary Fig. 4 shows a distribution $\kappa (k)=\frac{Q(k)}{N(k)}\sim {k}^{\lambda }$ where *Q*(*k*) is the probability of a new entrant connecting to an old firm of size *k*, *N*(*k*) is the number of firms with *k*, and *λ* is the exponent of preferential attachment^23^. Each exponent in 1994 and 2014 is about $\lambda \simeq 1.0\pm 0.1$ estimated by the least squares method with coefficient of determination *R* ^2^ ≥ 0.9 and error-bars show standard deviation, which is statistically consistent.

### Numerical analysis of selective M&A mechanism effects on firm-ecosystem

In order to better understand, we introduce simulation based on network evolution model and numerically analyze our ecosystem’s time evolution by using inter-firm business transactions networks. There are various types of models that focus on network evolution^9, 24–27^. Among them, Miura *et al*.^19^ introduced so-called MTT model and found that coagulation, namely M&A, is one of the important processes to reproduce distribution of transaction partners *k* that follows a power law consistent with that of Japanese inter-firm business transactions network (Supplementary Fig. 1). Furthermore, their model considers firm events such as new establishments and bankruptcies, and we decided to generalize MTT model to replace the merger kernel by the empirical form $K({k}_{a},{k}_{t})\propto {k}_{a}^{\alpha }{k}_{t}^{\beta }$ in equation (1).

Based on this revised model, we conducted Monte Carlo simulation to analyze time evolution of the ecosystem of business firms numerically (see Methods and Supplementary Fig. 5). We start with *N*_0_ = 100,000 firms without any business transactions and evolve the system by choosing one of the three events, new establishments, M&A and bankrupts, stochastically for 10,000,000 time steps where 8,000 steps corresponds to one year. Here, occurrence probabilities of new establishments, M&A, and bankrupts are 0.5, 0.1, and 0.4, respectively, estimated by the state of real ecosystem in Japan. The parameter-sets (*λ*, *α*, *β*) are empirically observed (1.0, 0.8, 0.6) and (1.0, 1.1, 0.7) for the year of 1994 and 2014, respectively (Fig. 2e,f). The difference of coagulation kernel causes a big difference in stability of systems as explained below.

**Figure 3:**
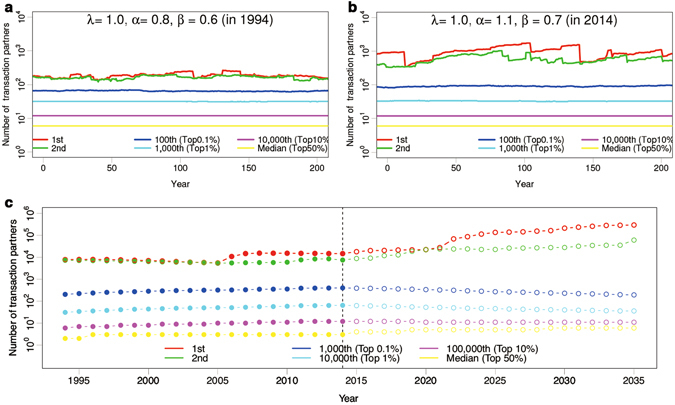
Simulated time evolution of number of transaction partners. Each mark shows size of 1st (red), 2nd (green), top 0.1% (blue), top 1% (light blue), top 10% (violet) and top 50% (yellow) in semilog plot. (**a**,**b**) Examples of simulated time series with initially *N*_0_ = 100,000 firms using empirically observed parameter-sets (*λ*, *α*, *β*) for the years of 1994 and 2014, respectively. (**c**) Time series of Japanese inter-firm business transactions network from 1994 to 2014, and simulated time evolution of that from 2015 to 2035 using the parameter-set of 2014.

We observed examples of simulated time series after 2,000,000 steps; 1st, 2nd, 100th (top 0.1%), 1,000th (top 1%), 10,000th (top 10%) and median (top 50%) to check a system’s stability. As for the year 1994 (Fig. 3a), the state of the system is almost stable. By contrast, the state is unstable in 2014 (Fig. 3b); there are larger fluctuations in 2014 than in 1994 because of the occurrences of dominant entities for every few decades. The number of transaction partners of the dominant company grows exponentially with the age of the firm on average (Supplementary Fig. 6c,i). Especially, focusing on a growth speed of nodes which have the most number, top degree nodes have a lead of 10 times over second-top degree nodes by 45 ± 22 years on average after disappearing ex-top degree nodes (Supplementary Fig. 7).

We hence numerically confirm that the selective M&A mechanism that prefers to merge with larger size of firms leads to significant concentration and the dominance of large entities, and a consequent suppress of growth of middle-sized firms (see Supplementary Information; A consequence suppress of growth of middle-sized firms). An example of abrupt changes in the number of transaction partners *k* of the dominant company is shown in Fig. 3c, which represents time evolution of real firms up to 2014. The firm that had second largest number of transaction partners (green marks) from 2000 to 2005 merged its 27 subsidiaries in 2006 and since then, it has kept the 1st seat (red marks). Figure 3c also shows a speculation of firm-ecosystem from 2015 to 2035 by using parameter-set in 2014, which the initial condition is the real network in 2014 with *N*_0_ = 1,132,629; 1st, 2nd, 1,000th (top 0.1%), 10,000th (top 1%), 100,000th (top 10%) and median (top 50%). Only two superordinates will continue growing.

## Discussion

We empirically find that our Japanese business firms have the specific property of the selective M&A mechanism that benefits large-sized firms growth. This means that there is an arbitrary “selection and concentration” process within our ecosystem, which leads to an imbalance to the latter. Within the real world Japanese data, we are also able to identify that the two separate dynamics present within M&A, namely the willingness of a company to merge, and its choice of partner, with the former having a higher influence than the latter. The increasing strength from 1994 to 2014 of the Cumulative Advantage mechanism that underpins the willingness to merge resulted in an acceleration of the inequality of income among companies as observed by the increase in the GINI index^28^ (Supplementary Fig. 8). Indeed the concentration of income within large companies is documented by other recent researches^21^, but no mechanism was explained. Therefore, importance must be given to the fact that strong selective and concentrative merger allows exclusive possession of the wealth to a mega-firm deriving the unstable fragile economy by its failure with potentially major negative consequences to the global economic network in the long timespan. We believe that our methods based on big data analysis and modeling can be added to basic scientific tools for stability analysis of complex ecosystems in the real world not limited to the business firms’ ecosystem.

## Methods

### Monte Carlo Simulation

As for our numerical analysis to estimate our ecosystem’s future behaviour, we conducted Monte Carlo simulation as follows;

- Start with *N*_0_ firms without any business transactions (*N*_0_ = 100,000 in Fig. 3a,b, and *N*_0_ = 1,132,629 in Fig. 3c).
- Choose one of the following three events stochastically. The occurrence probabilities of new establishments, M&A, and bankrupts are denoted by *r*_*n*_, *r*_*m*_, and *r*_*b*_, respectively.

#### New establishments

A new firm having four transaction partners is added; it is roughly consistent with the rate between number of firms and number of transaction partners. Besides, each transaction partner is connected to a firm chosen randomly following the preferential attachment rule with exponent *λ* (Supplementary Fig. 4).

#### M&A

Firms are randomly chosen following the merger kernel $K({k}_{a},{k}_{t})\propto {k}_{a}^{\alpha }{k}_{t}^{\beta }$ to choose an acquirer firm *a* and a target firm *t* (Fig. 2e,f). All the transaction partners connected to the target firm *t* are also rewired to the acquirer firm *a*.

#### Bankrupts

A randomly chosen firm is removed, along with all transaction partners connected to this firm. This is because age of firms follow exponential distribution; it is roughly consistent with the simple assumption that a firm disappears randomly following a Poisson process^19, 29^ (Supplementary Fig. 6a).

- Repeat **Step2** for 10,000,000 times.

We also illustrate our algorithm by schematic diagram in Supplementary Fig. 5b. The occurrence probabilities of new establishments, M&A, and bankrupts are denoted by *r*_*n*_, *r*_*m*_, and *r*_*b*_, respectively, satisfying ${r}_{n}+{r}_{m}+{r}_{b}=1$ (systems are quasi-stationally states when ${r}_{n}={r}_{m}+{r}_{b}$ in the number of firms). We here used ${r}_{n}=0.5$, ${r}_{m}=0.1$ and ${r}_{b}=0.4$ corresponding to the state of ecosystem in Japan (Supplementary Fig. 5c). In the case *N*_0_ = 100,000, 8,000 steps corresponds to one year which is estimated by matching the age of fimrs’ distribution (Supplementary Fig. 6a,g). In the case of simulation of Fig. 3c after 2015, the real network structure is used as the initial condition and the parameters are given by the real values measured in 2014. One year is given by 80,000 steps.

## Electronic supplementary material

Supplementary Information

Supplementary Video 1(a)

Supplementary Video 1(b)

Supplementary Video 1(c)
