# Supplementary material for: Appearance of Unstable Monopoly State Caused by Selective and Concentrative Mergers in Business Networks
Source: Sci Rep. 2017 Jul 11;7:5064. doi: 10.1038/s41598-017-05362-5 (PMC5506033; doi:10.1038/s41598-017-05362-5)
Supplement: Supplementary file 1 — Supplementary Information [file 41598_2017_5362_MOESM1_ESM.pdf]

Supplementary Information : Appearance of Unstable Monopoly State Caused by Selective and Concentrative Mergers in Business Networks

Hayato Goto[1,*], Eduardo Viegas[2], Henrik Jeldtoft Jensen[2,3], Hideki Takayasu[3,4], and Misako Takayasu[1,3]

[1]Department of Computational Intelligence and Systems Science, Interdisciplinary Graduate School of Science and Engineering, Tokyo Institute of Technology, 4259, Nagatsuta-cho, Yokohama 226-8502, Japan
[2]Centre for Complexity Science and Department of Mathematics, Imperial College London, SW7 2AZ, United Kingdom
[3]Institute of Innovative Research, Tokyo Institute of Technology, 4259, Nagatsuta-cho, Yokohama 226-8502, Japan
[4]Sony Computer Science Laboratories, 3-14-13, Higashi-Gotanda, Shinagawa-ku, Tokyo 141-0022, Japan
[*]goto.h.ac@m.titech.ac.jp

S1. Data source

Business practices in Japan are unique. When building trustworthy relationships or managing credit risk, Japanese people first tend to gather their business partners' detailed corporate information. Then, professional third-party organizations are used to search their partners' credit status. Teikoku Databank, Ltd is one of the largest corporate research providers in Japan; it has assessed the credit status of firms for 116 years. Their credit research reports include detailed information of the financial statements of firms, their history, business partners, management and banking transactions. The following types of data are used;

1. Inter-firm business transactions network data (which link the direction from consumers to suppliers in 2014). Each total number of firms and transaction partners is $1,132,629$ and $4,100,103$, respectively.

2. Firm sales' and number of employees' information data in 2014. Total number of firms is $1,452,381$.

3. M&A data (which link the direction from acquirers to targets, and have been stored from 1938 to 2014). Total number of events is $41,276$, which includes $41,276$ firms as acquirers and $60,464$ firms as targets.

Besides, as for data reliability, the Minister of Finance in Japan and the Statistics Bureau of Japan report that there are about 1,740,000 corporations with total annual sales about 1,378 trillion yen and 1,750,071 companies, respectively. Our database records 1,452,381 corporations with total annual sales about 1,343 trillion yen. Compared with these, our database keeps reliability by covering about 84% of total number of corporations and about 98% of total annual sales. We note that the Statistics Bureau of Japan simultaneously reports that the number of establishments in Japan is 5,779,072 in 2014. According to their explanation of terms, however, this number includes the number of branch offices or dummy companies. In this paper we use the terminology "firm" to represent "corporation" and "company" that all branches are considered to be aggregated to a head office.

S2. Scaling behaviour and Power laws

Various types of scaling laws are known for firms between number of transaction partners k, number of employees E and sales S as follows [1];

$$S \propto k^{1.3},\ S \propto E^{1.3},\ E \propto k^{1.0} \qquad (1)$$

We here reproduce those relations by our data in 2014. In Supplementary Figure 1a, we analyze relation between k (horizontal axis) and S (vertical axis). The value of k measured median over the bins of S is plotted in log-log scales, and the dashed-line shows an approximated power law with exponent 1.3 ± 0.1. Thus, we can confirm that scaling relations for S conditioned by k is expressed as $S \propto k^{1.3\pm0.1}$. In Supplementary Figure 2b and Supplementary Fig.2c, relations between S and E and between E and k at the median point are plotted in log-log scale, respectively. The relations for S conditioned by E and for E conditioned by k are $S \propto E^{1.3\pm0.1}$ and $E \propto k^{1.0\pm0.1}$, respectively same as the results of previous study in Equation (1).

Moreover, we find new scaling laws with number of ancestors A; scaling relations between k and A, between E and A, and between S and A are plotted in Supplementary Fig.2d,e,f, respectively.

$$k \propto A^{1.4\pm0.1},\ E \propto A^{1.4\pm0.1},\ S \propto A^{1.8\pm0.1} \qquad (2)$$

All these quantities additionally follow power law cumulative distributions with exponents 1.4, 1.0 and 2.4, respectively (Supplementary Fig.1).

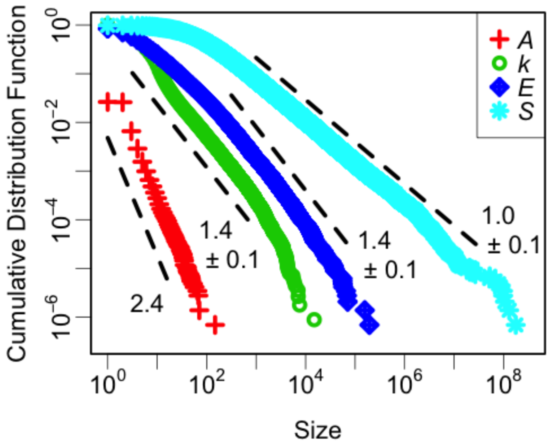

Supplementary Figure 1: Cumulative distributions of firms' various sizes in log-log scale plot. Red crosses, green circles, blue rhombuses and light blue asterisks show ancestors A, transaction partners k, employees E and sales (million yen) S, respectively. Each dashed line shows an approximated power law distribution.

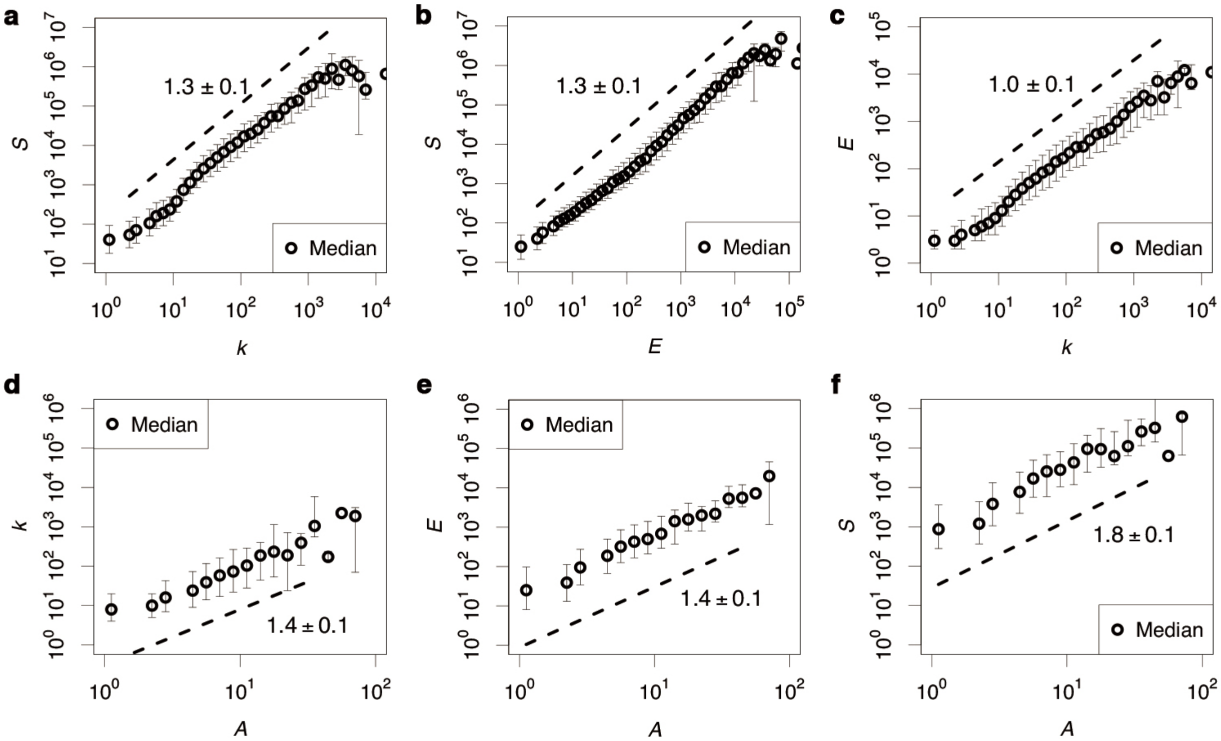

Supplementary Figure 2: Scaling laws between pairs of number of transaction partners k, number of employees E, sales S, and number of ancestors A. The value of horizontal axis measured median over the bins of a vertical axis is plotted in log-log scales. Each dashed-line shows an approximated power law slope. **a** Horizontal and vertical axes are k and S, respectively; $S \propto k^{1.3\pm0.1}$. **b** Horizontal and vertical axes are E and S, respectively; $S \propto E^{1.3\pm0.1}$. **c** Horizontal and vertical axes are k and E, respectively; $E \propto k^{1.0\pm0.1}$. **d** Horizontal and vertical axes are k and A, respectively; $k \propto A^{1.4\pm0.1}$. **e** Horizontal and vertical axes are E and A, respectively; $E \propto A^{1.4\pm0.1}$. **f** Horizontal and vertical axes are S and A, respectively; $S \propto A^{1.8\pm0.1}$.

S3. Growth rate of firms' size after M&A

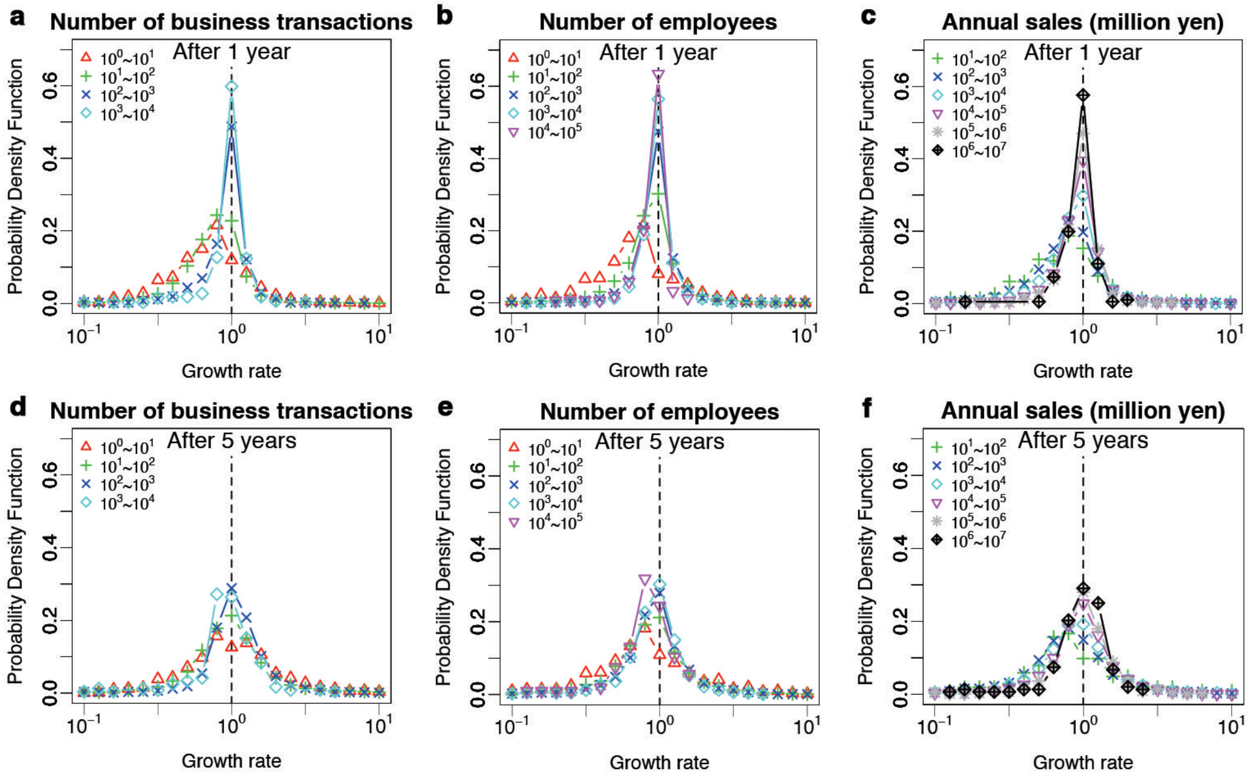

Supplementary Figure 3: Probability density distribution of growth rate of firms' size which are compared a merged entity after a year (**a,b,c**) and five years (**e,f,g**) with the sum of an acquirer firm and target firms for each volume such as number of transaction partners k (**a,d**), number of employees E (**b,e**) and sales S (**c,f**). Red squares, green plus-marks, blue crosses, light blue rhombuses, purple downward triangles, grey astarisks and black squares with cross show each firm-size range from 10^0 to 10^1, from 10^1 to 10^2, from 10^2 to 10^3, from 10^3 to 10^4, from 10^4 to 10^5, from 10^5 to 10^6 and from 10^6 to 10^7, respectively.

S4. Preferential attachment for established firm

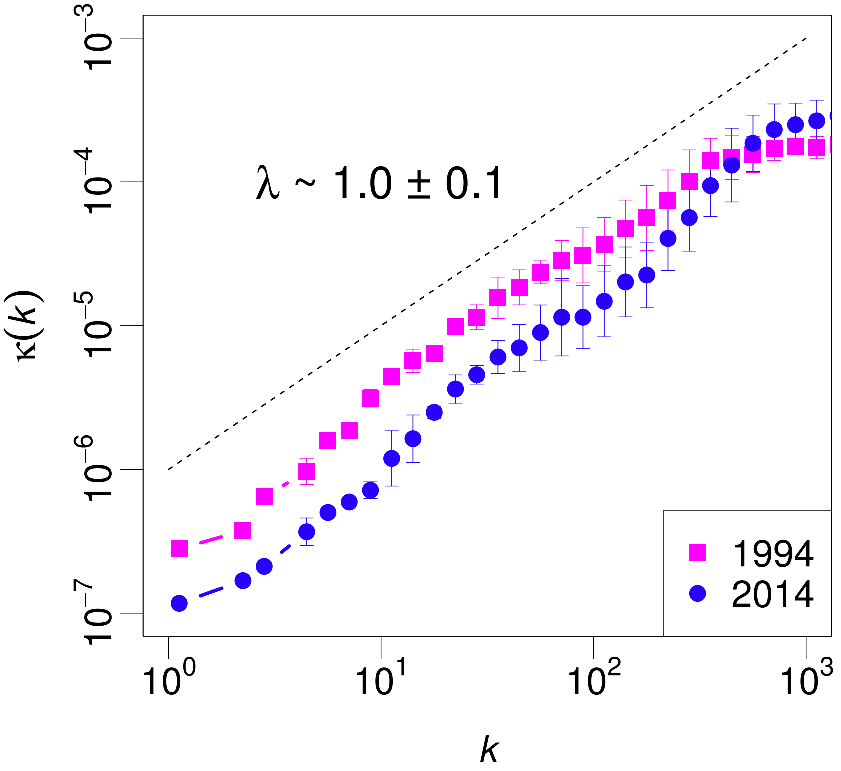

Supplementary Figure 4: Scaling relation of the rate of preferential attachment as a function of k, $\kappa(k)$, in log-log scale plot. Pink squares and blue circles show the mean of $\kappa(k)$ for 1994 and 2014, respectively.

S5. Illustration of our simulation model

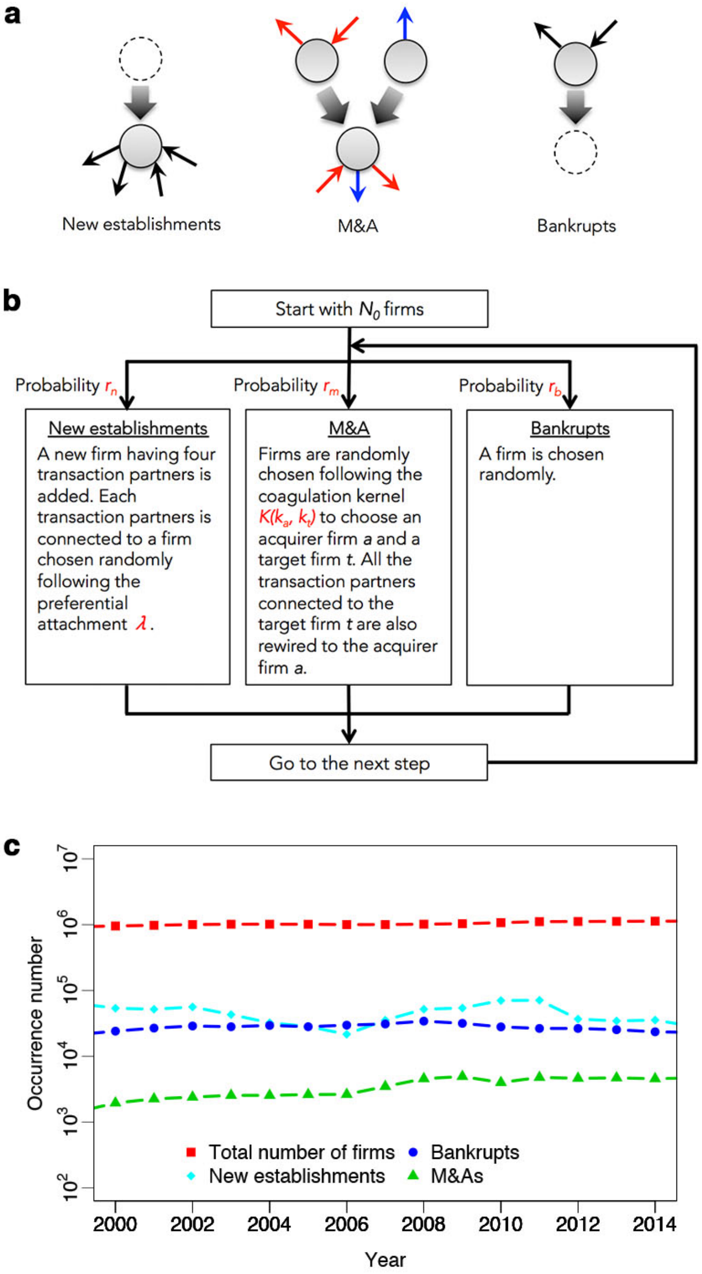

Supplementary Figure 5: **a** Schematic figures showing the three basic processes for firms; M&A, new establishments, and bankrupts. **b** A flow chart showing the time step of our simulation model. **c** Time series of actual number of firms (red), new establishments (light blue), M&A (green) and bankrupts (blue) in Japan. Number of firms is almost stable, and the occurrence ratio between M&A and bankrupts is about 0.1 : 0.4. We therefore used the occurrence probabilities of new establishments (r_n), M&A (r_m), and bankrupts (r_b) are 0.5, 0.1, and 0.4, respectively.

S6. Validity verification

In Supplementary Figure 6, we compare empirical analysis results (a,b,c) with simulation results (d,e,f for $N_0 = 10,000$ and g,h,i for $N_0 = 100,000$) from the viewpoints of distribution of age of firms [2, 3], distribution of number of transaction partners [4], and relation between age of firms and average number of transaction partners [2].

In Supplementary Figure 6a, cumulative distribution of age of firms since founded is plotted in semilog scale showing that it is well characterized by an exponential function, $exp(-t/\tau)$, where $\tau \simeq 55$ years is the characteristic decay time, and in Supplementary Fig.6d and g the corresponding distribution by simulation are plotted. These exponential distributions are roughly consistent with the simple assumption that a firm disappears randomly following a Poisson process. In comparison of time constants between the empirical observation and the simulation result, one year corresponds to about 800 for $N_0 = 10,000$ and $8,000$ for $N_0 = 100,000$ steps.

In Supplementary Figure 6b, cumulative distribution of number of transaction partners is plotted in log-log scale, and its corresponding figure by simulations are plotted in Supplementary Fig.6e and h. Both graphs are well characterized by power laws with exponent 1.4.

Lastly, in Supplementary Figure 6c, we observe the relation between the number of transaction partners and the age of firms since founded. The value of number of transaction partners averaged over the bins of age of firms is shown in the semilog plot; the result implies that the number grows exponentially with the age, $exp(6.0x10^{-3}t)$. A consistent result is confirmed by simulation in Supplementary Fig.6f and i.

As for growth speed of nodes which have the largest link number, we calculate average necessary time that the top degree node has a lead of 10 times over the second-top degree node after disappearing an ex-top degree node. Probability density distribution of the time is plotted in semilog scale in Supplementary Fig.7 by 200 attempts. Hence, after disappearance of ex-dominant firms, there would be a potential for new dominant firms' appearance about 45 ± 22 years on average.

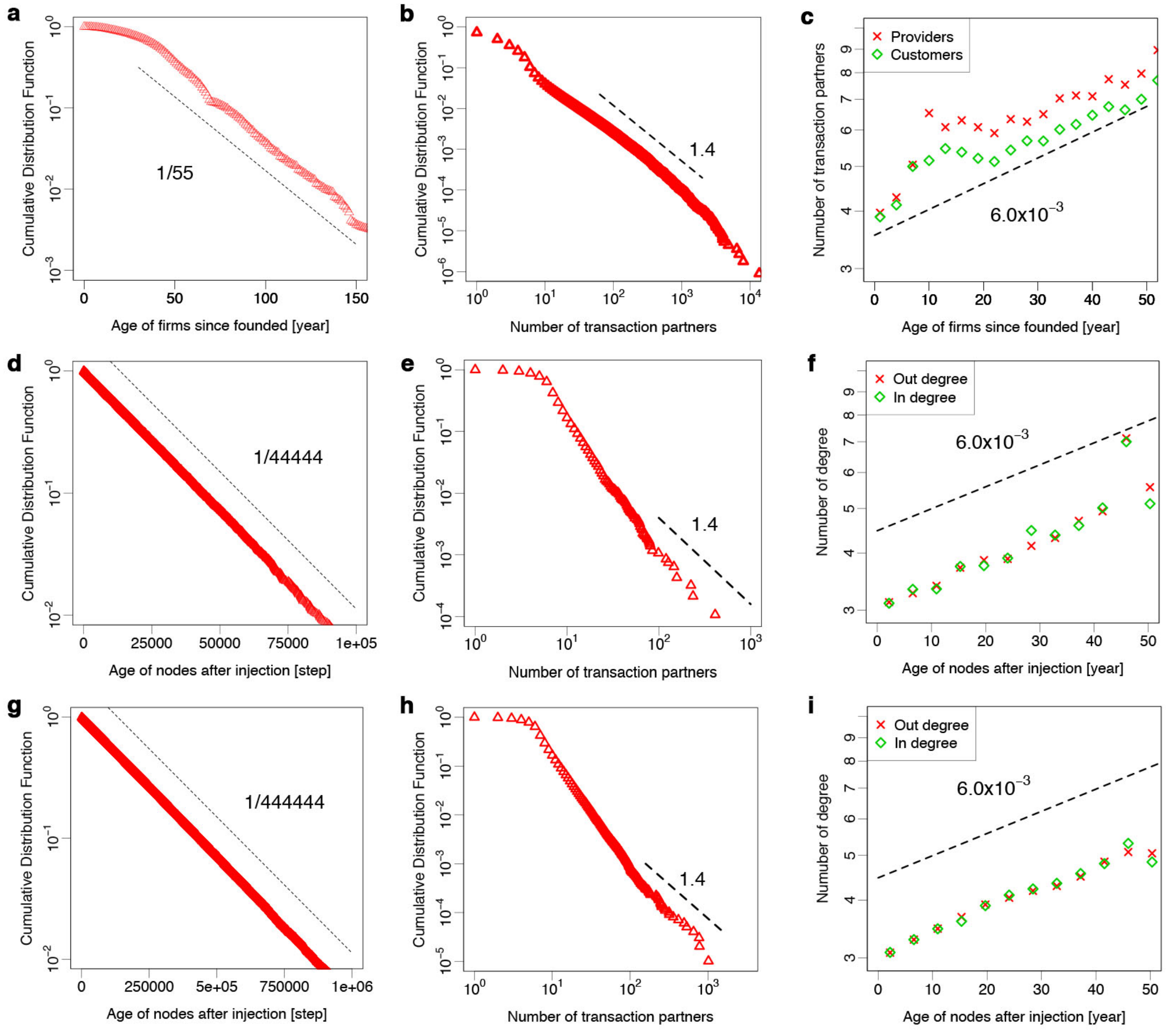

Supplementary Figure 6: **a,b,c** Empirical analysis results in 2014. Monte Carlo simulation results with $N_0 = 10,000$ (**d,e,f**) and $N_0 = 100,000$ (**g,h,i**) using empirically observed parameter-sets $(\lambda, \alpha, \beta) = (1.0, 1.1, 0.7)$ adjusted for the years of 2014. **a,d,g** Cumulative distribution of age of firms since founded in semilog plot. Each dashed line shows an approximated exponential distribution with slope $1/55$, $1/44,444$ and $1/444,444$, respectively. **b,e,h** Cumulative distribution of number of transaction partners in log-log plot. Each dashed line shows an approximated power law distribution with slope 1.4. **c,f,i** Relation between age of firms since founded and average number of transaction partners in a semilog plot. Red crosses for in degree and green squares for out degree; the dotted line indicates an exponential growth equivalent to $exp(6.0x10^{-3}t)$.

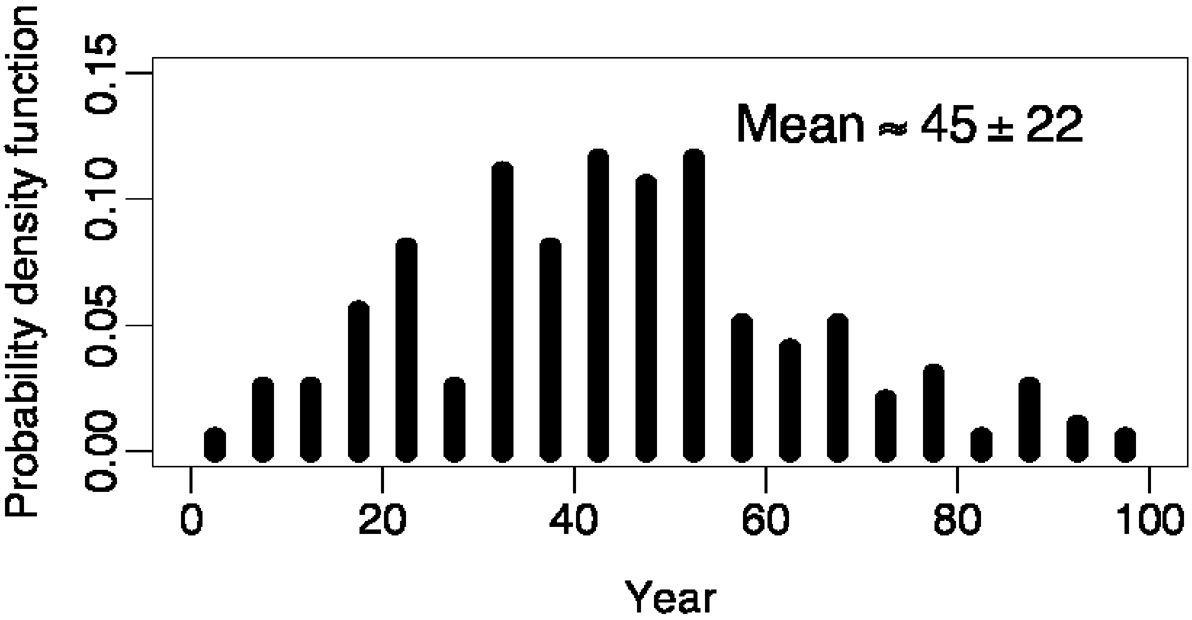

Supplementary Figure 7: Probability density distribution of a necessary time that the top degree node has a lead of 10 times over the second-top degree node after disappearing an ex-top degree node by 200 numerical attempts, where about 45 ± 22 years on average.

S7. A consequence suppress of growth of middle-sized firms

We observe cumulative distributions of number of transaction partners corresponding to simulated time evolution in Fig.3. Supplementary Video 1(a) and (b) show results with $N_0 = 10,000$ by using parameters which are given by the real values measured in 1994 and 2014 in log-log scales, respectively. For better understanding, besides, each firm-size range is defined by using number of transaction partners as follows; small-sized firms are from 10^0 to $10^{1.5}$ (black), middle-sized firms are from $10^{1.5}$ to 10^3 (blue), and large-sized firms are over 10^4 (red).

As for the case in 1994, there are little fluctuations in small and middle-sized firms in every period. Compared with this, in 2014, the number of midlle-sized firms decreases with appearance of large-sized firms while there is almost no effect on small-sized firms. These results suggests that the selective M&A mechanism that prefers to merge with larger size leads to significant concentration and the dominance of large entities, and a consequent suppress of growth of middle-sized firms.

Supplementary Videos 1(c) additioanlly shows one of example results with $N_0 = 10,000$ by using $\lambda = 1.0,\ \alpha = 1.4,\ \beta = 0.8$ that the tendency of the selective M&A mechanism becomes stronger than 2014. It is easier to find the consequent decrease of middle-sized firms by the selective M&A mechanism.

Supplementary Video 1: Cumulative distributions of number of transaction partners in log-log scales corresponding to simulated time evolution by using each parameter. Each firm-size range is defined as follows; small sized-firm is from 10^0 to $10^{1.5}$ (black), middle is from $10^{1.5}$ to 10^3 (blue), and large is over 10^4 (red). **(a)** A case that takes into account of the parameters, which are given by the real values measured in 1994 (Fig.2e). **(b)** A case that takes into account of the parameters, which are given by the real values measured in 2014 (Fig.2f). **(c)** A case that takes into account of the parameters, which are $\lambda = 1.0,\ \alpha = 1.4,\ \beta = 0.8$ that the tendency of the selective M&A mechanism becomes stronger than 2014.

S8. GINI index

GINI index is one of measurements that reflects the deviation of inequality in a distribution [5]. It is mainly used to evaluate family income distribution in a country. We here use the index to explain the inequality of income among companies. As observed in Supplementary Fig.8, the Cumulative Advantage mechanism, which underpins the willingness to merge, has made the inequality among companies stronger.

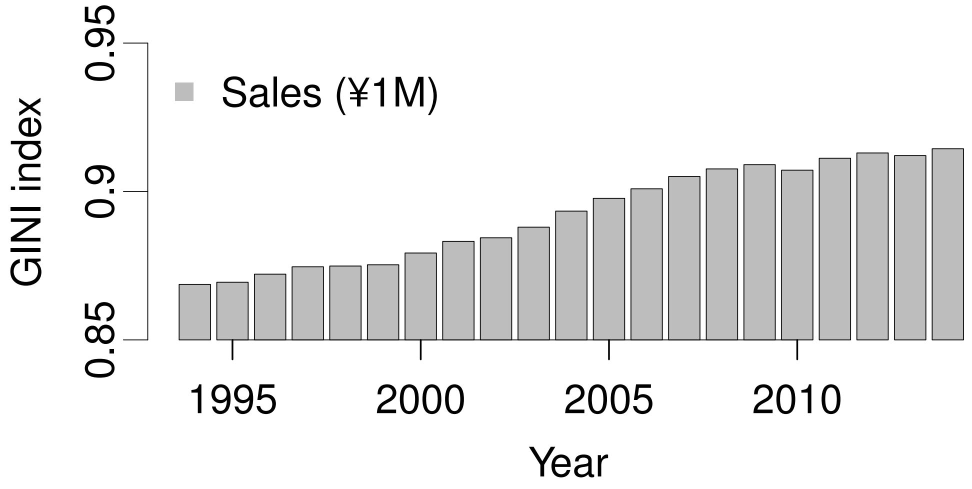

Supplementary Figure 8: Time series of Japanese firms' GINI index calculated by annual corporation sales from 1994 to 2014. It has slightly increased by 5% from 1994.

References

[1] Watanabe, H., Takayasu, H. & Takayasu, M., Relations between allometric scalings and fluctuations in complex systems: The case of Japanese firms, *Phys. A*, **392**, 741-756, (2013).

[2] Miura, W., Takayasu, H. & Takayasu, M., Effect of coagulation of nodes in an evolving complex network, *Phys. Rev. Lett.*, **108**, 168701, (2012).

[3] Goto, H., Takayasu, H. & Takayasu, M., Empirical Analysis of Firm-Dynamics on Japanese Interfirm trade Network, *Social Modeling and Simulation plus Econophysics Colluquium 2014, Proc. Int. Conf.*, 195-204, (2014).

[4] Takayasu, M. et al., Massive Economics Data Analysis by Econophysics Methods-The case of companies' network structure, *Earth Simulator Center Report*, **263**, (2008).

[5] Gini, C., Variabilit e mutuabilit. contributo allo studio delle distribuzioni e delle relazioni statistiche, *Tipogr. di Cupini*, (1912).
